# Does self-stigma impact blood glucose control in people with type 1 diabetes?

**Published:** 2023-02

**JANUARY 25, 2023** - Individuals with chronic medical conditions may experience self-stigma, or negative beliefs, emotional reactions, and behaviors towards themselves as a result of their illness. New research published in the Journal of Diabetes Investigation found a link between self-stigma and glycated hemoglobin (HbA1c)—a marker of blood glucose levels—in adults with type 1 diabetes.

The study included 109 adults in Japan with type 1 diabetes who completed questionnaires that generated scores based on a self-stigma scale. Although the findings support a link between self-stigma and sub-optimal HbA1c, additional studies are needed to show whether this is a causal relationship.

“We focused on this issue through clinical experiences with people with type 1 diabetes, whose glycemic management improved markedly by social supports of eliminating diabetes-related stigma. Although the finding of an association between self-stigma and HbA1c is significant, further longitudinal research is required to determine whether self-stigma leads to sub-optimal HbA1c,” said corresponding author Yukiko Onishi MD, PhD, of the Institute of Medical Science, Asahi Life Foundation, in Tokyo. “This research does support and highlight the importance of eliminating self-stigma when treating people with type 1 diabetes.”

*Link to Study: https://onlinelibrary.wiley.com/doi/10.1111/jdi.13963*

*Full citation: “Association of self-stigma with glycated hemoglobin: A single-center, cross-sectional study of adults with type 1 diabetes in Japan.” Shoko Hamano, Yukiko Onishi, Yoko Yoshida, Toshiko Takao, Tazu Tahara, Takako Kikuchi, Toshiko Kobori, Tetsuya Kubota, Masahiko Iwamoto, Masato Kasuga. Cox, Michael Olivier. J. Diabetes Investig; Published Online: November 23, 2022 (DOI: 10.1111/jdi.13963).*

*Copyright © 2019 The Cochrane Collaboration. Published by John Wiley & Sons, Ltd., reproduced with permission.*

